# Progress and Prospects of Polymer/One-Dimensional Nanoclay Superabsorbent Composites

**DOI:** 10.3390/polym17050669

**Published:** 2025-02-28

**Authors:** Haifeng Xing, Xiangyu Liu, Qingdong He, Wenbo Wang

**Affiliations:** 1 College of Resources and Environmental Sciences, Inner Mongolia Agricultural University, Hohhot 010010, China; 2 College of Chemistry and Chemical Engineering, Inner Mongolia University, Hohhot 010030, China; lxy13257244737@163.com (X.L.); 18847744604@163.com (Q.H.)

**Keywords:** superabsorbent, one-dimensional nanoclay, palygorskite, sepiolite, halloysite, water absorbency

## Abstract

Superabsorbent materials (SAMs), featuring a three-dimensional (3D) hydrophilic polymer network, can absorb and retain water up to thousands of times their own weight, even under pressure. This makes them indispensable in various fields, including hygiene products and agriculture. The water absorption capacity of SAMs is influenced by the presence of hydrophilic groups and a swellable network structure. To optimize performance, one must adjust the types and concentrations of functional groups. Additionally, changes in the density and regularity of the polymer network are necessary. Significant performance improvements are limited by inherent challenges in modifying polymer chains or networks. To enhance performance, researchers focus on manipulating the components and structure of the polymer network. Effective water retention requires the network to fully expand while maintaining its strength. Incorporating nanoparticles, especially one-dimensional (1D) nanoclays, minimizes chain entanglement and prevents network collapse during drying. This approach effectively addresses the above challenges. Upon swelling, these nanoparticles improve hydrogen bonding within the polymer network, significantly boosting the performance of SAMs. Nanoclays are abundant natural silicates found in various nanostructures like nanorods, nanofibers, and nanotubes. These nanoclays contain reactive silanol groups that form strong hydrogen bonds with polymer chains. This aids in network formation and reduces costs. Advances in synthesis and structural control have facilitated the development of versatile 1D nanoclay-based SAMs. This paper reviews the structure, characteristics, and applications of such materials and proposes future research directions aimed at developing higher-performance clay-based SAMs.

## 1. Introduction

Water-absorbing materials have become an indispensable component in daily production and life. Superabsorbent materials (SAMs) outperform traditional absorbents due to their superior water absorption and retention capabilities. These materials can quickly absorb large quantities of water and retain it even when heated or under pressure [1,2,3,4]. Since their introduction, SAMs have seen rapid advancement in design and application, achieving commercialization [5]. Their unique properties have led to widespread use in various sectors such as agriculture and forestry [6,7,8], hygiene products [9,10], wastewater treatment [11,12,13,14], drug delivery systems [15], and nutrient slow-release [16,17]. Advances in material science and chemistry continue to drive the development of SAMs through specialized structures, compositions, and performance characteristics, expanding their application fields.

The aim of this continuous pursuit is to enhance the performance of SAMs while reducing costs. Water absorption mechanisms can generally be categorized into those of physical adsorption and chemical adsorption [18]. Traditional water absorbents primarily rely on capillary action for physical adsorption, which leads to easy saturation and the loss of absorbed water under slight pressure. In contrast, SAMs consist of three-dimensional (3D) polymer networks formed through polymerization and chemical crosslinking or physical entanglement. They involve both physical and chemical adsorption [19]. After absorption, water molecules are confined within a fixed network space formed by chemical or physical crosslinks, measuring 1–10 nanometers in length. The physical and chemical adsorption within the 3D macromolecular network facilitates the swift movement of water molecules into the network.

**Figure 1:**
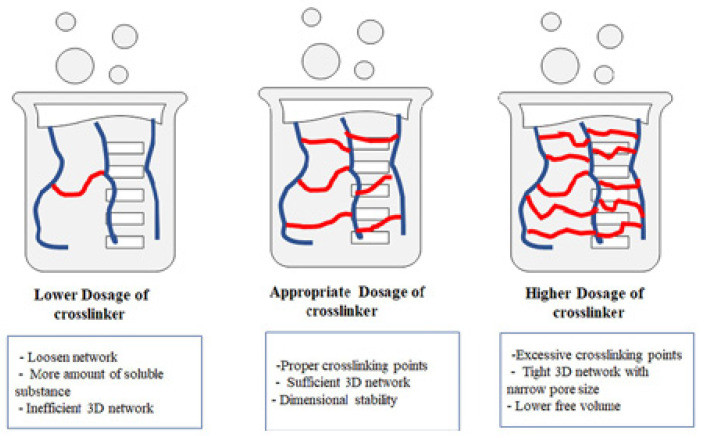
Crosslinking network structure with different crosslinking density [20]. Reproduced with the permission of the publisher.

Before water absorption, the polymer network is a solid bundle. Upon contact with water, the hydrophilic ionic groups of the SAMs dissociate, fixing anions along the macromolecular chain while surrounding them with mobile cations. As shown in Figure 1 [20], different crosslinking densities result in varied typical crosslinked networks. During absorption, electrostatic repulsion between anions causes the polymer network to expand. This creates an osmotic pressure difference, driving more water molecules into the network. As water absorption increases, the osmotic pressure difference approaches zero, while the elastic contraction force of the polymer chains gradually rises. When the elastic contraction force balances the electrostatic repulsion, expansion equilibrium is achieved. The polymer backbone in the 3D crosslinked network includes carboxyl, amino, and hydroxyl groups. These groups enable it to absorb and retain large quantities of water or aqueous solutions without dissolving. This leads to increased network entropy and rapid expansion of the SAMs. Under stable network structure conditions, the larger the 3D network space, the higher the water absorption rate.

Incorporating organic components like humic acid or inorganic constituents like clays into the SAMs network modifies the network structure. This impacts the water absorption capacity, gel strength, salt resistance, and thermal stability of the SAMs [21]. The main chain of SAMs can be starch, cellulose, or synthetic polymers containing numerous hydrophilic groups like hydroxyl, carboxyl, amide, and sulfonic acid groups [22]. As environmental awareness grows and there is an increasing emphasis on material eco-friendliness, researchers aim to improve SAM performance, reduce costs, and lower the percentage of petroleum-based monomers to enhance safety. Utilizing naturally available resources such as polysaccharides, biomass, and clays has emerged as the optimal approach to improving the safety and cost-efficiency of materials [23,24,25,26]. Introducing natural polymers can transform the polymer chain structure from simple copolymerization to graft copolymerization, enhancing water absorption and environmental friendliness [27]. Adding inorganic components can prevent excessive polymer chain entanglement and improve network structure through hydrogen bonding or van der Waals forces. This significantly enhances the overall performance of SAMs [28,29]. Studies confirm that adding inorganic clay particles improves water retention, network strength, and thermal stability [30,31]. Consequently, inorganic-mineral-based SAMs have attracted significant research interest. Introducing one-dimensional (1D) nanoclay into hydrophilic polymer networks to form organic–inorganic composite structures is an ideal strategy for overcoming the limitations of pure organic polymer materials [32]. This paper summarizes the structure of 1D nanoclays and their application for fabrication of SAMs.

## 2. Structures and Characteristics of 1D Nanoclay

Clay minerals are naturally occurring silicates with unique morphologies. They have been widely used in various fields, including the chemical industry, environmental remediation, composite materials, and agriculture [33,34,35,36,37,38]. The 1D nanoclays, such as palygorskite (also called as attapulgite) (Pal) nanorods, sepiolite (SPT) nanofibers, and halloysite (HSY) nanotubes, are key members of the clay family. These materials can adsorb various molecules or ions due to their high ion exchange capacity, large specific surface area, and functional surface groups [39]. Additionally, they can absorb water and nutrients, making them suitable for improving water and fertilizer retention in sandy soils [40]. Clay minerals with superior surface activity are compatible with polymers and can act as reinforcing agents. This improves the strength and regularity of polymer networks, thereby enhancing their performance [41,42]. Thus, 1D nanoclay shows different advantages in improving the performance of polymer materials, such as mechanical strength, UV shielding performance, and water resistance [43]. The structural characteristics of various 1D clay minerals will be introduced in detail.

### 2.1. Pal Nanorods

**Figure 2:**
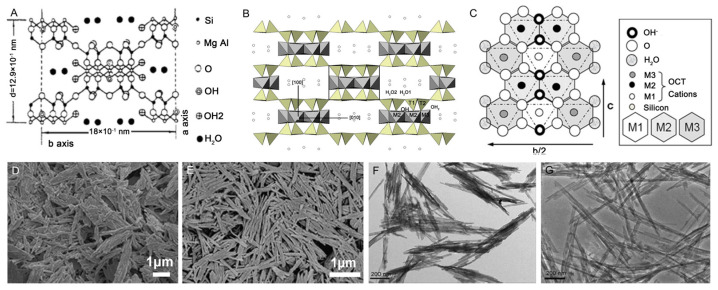
(**A**) The crystal structure (001 plane projection) and basic structure parameters [44]; (**B**) the basic structure of discontinuous tetrahedral sheet and continuous tetrahedral sheet, as well as the structural hydroxyl groups, coordination water, and zeolite water [53]; (**C**) the (100) plane of palygorskite, showing the M1, M2, and M3 sites in the octahedral layer [47,48]; SEM image of (**D**) natural Pal and (**E**) disaggregated Pal [54]; TEM image of (**F**) natural Pal and (**G**) disaggregated Pal [55]. Reproduced with the permission of the publisher.

Pal is a magnesium-rich aluminum silicate clay mineral with a 2:1 layered chain structure and a natural nanorod morphology. Its theoretical formula is Si_8_Mg_5_O_20_(OH)_2_(H_2_O)_4_·4H_2_O [44,45,46], and it features nanochannels measuring 0.37 nm × 0.64 nm along with active surface groups like Si–OH and Al–OH (Figure 2) [47,48,49]. The crystal structure of Pal consists of SiO_4_ tetrahedral layers that repeat every four units. Their apical oxygens link to Mg–O or Al–O octahedra, forming ribbons with a 2:1 layer structure. These ribbons are interconnected by Si–O–Si bonds, creating its distinctive layered chain framework. Ideally, Pal should be a trioctahedral mineral, with all octahedral positions occupied by Mg^2+^ ions. However, due to isomorphic substitution, some trivalent cations such as Al^3+^ and Fe^3+^ can replace Mg^2+^ at the octahedral sites, leading to dioctahedral or intermediate structures [50,51,52]. The nanorods of Pal (Figure 2D–G) interact with polymer matrices through their active surface groups, effectively modulating the polymer’s network structure. This interaction has made Pal widely applicable in polymer composites, enhancing physico-chemical properties and performance of materials.

### 2.2. SPT Nanofiber

**Figure 3:**
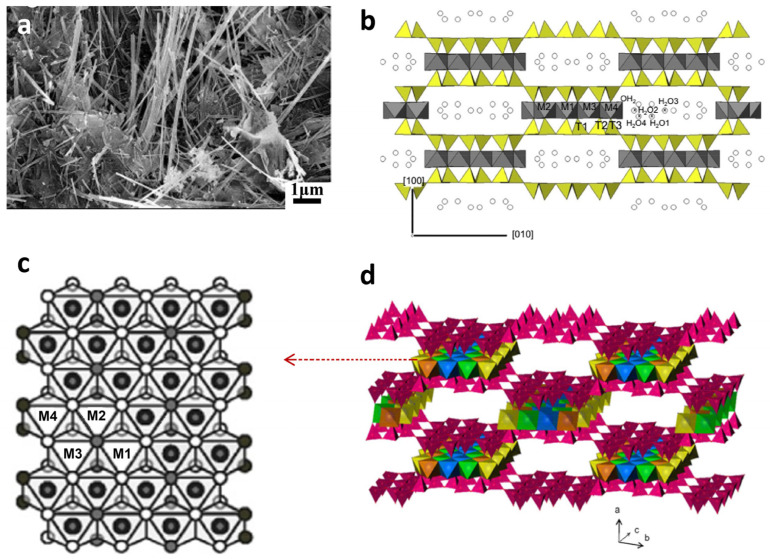
(**a**) Representative SEM-FEG image of sepiolite from Finland [56]; (**b**) projection of monoclinic sepiolite structure along the [001] direction [53]; (**c**) octahedral layer of SPT [65]; (**d**) the crystal structure of primitive sepiolite: silica oxygen tetrahedra appear red, while Mg(II) cations show different colors depending on their position in the octahedral sheet [72], in which the red arrow denotes the details of the octahedral sheet. Reprinted with permission of the corresponding publishers.

SPT is a fibrous, magnesium-rich silicate clay mineral with a layer-chain crystal structure. This structure comprises two tetrahedral silica units bound to a central octahedral magnesium sheet, leading to a fibrous morphology that can extend several micrometers in length (Figure 3a) [56,57]. Its structural formula is Mg_8_Si_12_O_30_(OH)_4_(OH_2_)_4_·8H_2_O, and it features uniform nanochannels measuring 1.06 nm × 0.37 nm (Figure 3b,d) [53,58]. The outer surface of SPT is rich in silanol groups (Si–OH). The coordination of octahedral cations is completed by OH_2_ molecules [59,60,61,62]. During SPT formation, octahedral Mg(II) cations are often partially substituted by Al(III) and/or Fe(III), leading to structural negative charges and defects. This substitution results in a high content of exchangeable cations within the nanochannels to balance these negative charges. Studies indicate that the MgO content in SPT varies widely, ranging from 30.6% to 18.6% [63]. Bailey also proposed an adjusted structural formula for SPT: (Mg_8-y-z_R_3+y_□_z_)(Si_12-x_R_3+x_)O_30_(OH)_4_(OH_2_)_4_R^2+^_(x-y+2z)_/_2_(H_2_O)_8_, where R^3+^ represents Al(III) or Fe(III), and □ indicates a vacancy [64]. The distribution of octahedral cations within SPT crystals can be described by four sites: M1, M2, M3, and M4 (Figure 3c) [58,65]. Typically, Mg(II) cations favor the M4 position, whereas R cations can occupy M1, M2, and M3 [65]. Due to its unique properties, SPT has found applications in adsorption [66,67], catalysis [68], polymer composites [69,70], and even animal feed [71]. Research into the use of SPT for developing hydrophilic polymer composites has highlighted its potential and promising prospects.

### 2.3. HSY Nanotubes

**Figure 4:**
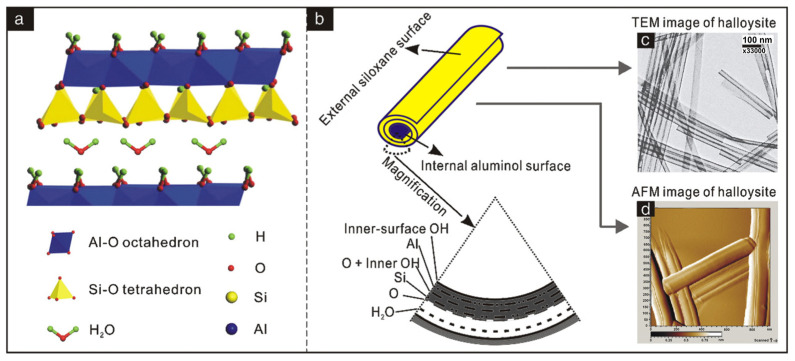
Schematic diagram of (**a**) crystalline structure of halloysite-(10 Å), (**b**) structure of HSY particle, (**c**,**d**) TEM and AFM images of HSY [74]. Reprinted with permission from Elsevier.

Halloysite (HSY) is a 1:1-type octahedral clay mineral, characterized by its nano-tubular morphology and association with the kaolinite group (Figure 4). The mismatch between the oxygen-sharing tetrahedral and octahedral sheets in its structure causes the nanolayer to curl [73,74]. HSY’s structural formula is Al_2_(OH)_4_Si_2_O_5_·nH_2_O, making it chemically similar to kaolinite. However, HSY has unit layers that are separated by a single layer of water molecules. HSY nanotubes typically range from sub-micron to several microns in length, sometimes exceeding 30 μm [75]. They have an outer diameter of approximately 30 to 190 nm and an inner diameter of about 10 to 100 nm [76]. This unique tubular structure makes HSY suitable for a variety of applications, including drug delivery systems [77,78], adsorption [79,80,81], catalysis [82,83,84], slow-release carriers for chemicals [85,86], and polymer composites [87,88,89]. HSY can be modified with ions or organic molecules to improve its surface properties or compatibility with other matrices [90]. This modification offers a new approach to creating superior nanocomposites for various applications.

## 3. Comparison of 1D Nanoclay with Other Clays in SAMs

The role of clays in SAMs depends on the type of polymer matrix used. For the same polymer matrix, the properties of the resulting SAMs depend on the type and structure of the clay. Therefore, it is necessary to compare different types of clays in the case of the same polymer matrix. Tragacanth gum-g-poly(AA-co-NHA)/clay SAM was prepared by grafting acrylic acid (AA) and N-hydroxymethacrylamide (NHA) onto gum tragacanth (GT) via solution polymerization, with an equal amount of various clays added. The effects of different clay types on the structure, morphology, and stability of the resulting SAMs were then investigated. For SAMs containing 3% Pal, kaolinite, and montmorillonite, their maximum water absorbency reached 1180, 819, and 525 g/g in deionized water; 206, 192, and 170 g/g in tap water; and 95, 82, and 62 g/g in a 0.9% NaCl solution, respectively. This indicates that Pal (3%) is more effective in improving water absorbency in the studied polymer system [91]. In addition, the SAM with 3% Pal also shows better water retention capacity. For another system (wheat bran (WB)-g-PAA), the role of different clay minerals (e.g., Pal, diatomite, and kaolinite) in improving the performance of SAMs was compared [92]. Different clays incorporated into the system resulted in SAMs with distinct structures and performances. In distilled water, their equilibrium water absorbency followed this order: WB-g-PAA (548 g/g) > WB-g-PAA/Pal (513 g/g) > WB-g-PAA/diatomite (506 g/g) > WB-g-PAA/kaolinite (437 g/g). Additionally, urea loading percentages were as follows: 81 wt% (WB-g-PAA/Pal) > 73 wt% (WB-g-PAA/diatomite) > 63 wt% (WB-g-PAA/kaolinite). These results indicate that the effect of clay on the performance of SAM does not have a universal pattern and is dependent on polymer matrix, and that 1D clay is more effective in improving SAMs.

## 4. Polymers/1D Nanoclay SAMs

Although organic SAMs exhibit excellent water absorption performance, there are still problems such as limited strength, poor salt resistance, and high cost. Principally, organic SAMs possess a flexible expansion network that becomes elastically flexible upon absorbing water molecules. Therefore, introducing rigid nanoparticles into this network is expected to improve its network structure and components, further enhancing its performance. The interaction between clays of different structures and the polymer matrix varies, leading to significant differences in the properties of the resulting SAMs. So far, a large number of SAMs have been synthesized by combining 1D nanoclay with synthetic polymer matrices, natural polymers, and their grafted polymer matrices, enriching the family of SAMs.

### 4.1. Synthetic Polymers/1D Nanoclays SAMs

The hydrophilic synthetic polymers are mainly prepared from vinyl monomers through free radical polymerization and crosslinking reactions. Key monomers for SAM synthesis include AA, acrylamide (AM), acrylonitrile (AN), and 2-acrylamido-2-methylpropanesulfonic acid (AMPS). These contain hydrophilic groups like carboxylic acids, amide groups, hydroxyl groups, amines, imides, and sulfonic acid groups. These groups make the polymer network highly hydrophilic, and the polymer chains can be expanded when in contact with water.

Palygorskite (also called as attapulgite) (Pal) is a silicate clay mineral with a distinctive nanorod crystalline morphology, featuring numerous active silanol groups on its surface that facilitate the formation of nanocomposites when combined with polymer matrices [93]. Researchers have developed a range of synthetic polymer/Pal SAMs through copolymerization and crosslinking reactions with hydrophilic monomers such as AA, AM, and AMPS, evaluating their performance extensively (Table 1) [94,95,96,97,98,99,100,101,102,103,104,105,106,107,108,109,110,111]. Research has especially concentrated on SAMs made from poly(acrylic acid) (PAA) and poly(acrylamide) (PAM) combined with natural Pal clay, as well as Pal modified through acid, heat, salt, or organic treatments. Among these, the composite made from PAM and organically modified Pal exhibits exceptional water absorbency, reaching up to 2800 g/g in deionized water and 121 g/g in a 0.9 wt.% NaCl solution [94]. The polymerization process involves the initiator ammonium persulfate (APS) decomposing into sulfur oxygen free radicals, which initiate the polymerization by attacking the vinyl groups of the monomers, leading to chain propagation. Crosslinkers then link the polymer chains into a network structure. During this process, while some hydroxyl groups on the Pal surface may participate in the reaction, most Pal particles are physically incorporated into the polymer network [95]. The incorporation of Pal improves the surface roughness of SAM, which facilitates enhancement of the water absorption capacity and rate. To broaden the application domain of these minerals, researchers have also investigated the swelling behavior and water retention of SAMs prepared from AA/AM copolymers and acid-modified Pal under ultrasonic treatment and varying pH conditions [112]. Findings show that an optimal amount of Pal can significantly enhance adsorption capacity. With ultrasonic power set at 200 W and a Pal content of 10%, the SAM achieves peak water absorbencies of 1257.54 g/g in distilled water and 209.45 g/g in a 0.9 wt% NaCl solution, maintaining high absorbency across a pH range of 5–9. This indicates that incorporating Pal can markedly improve the water absorption and retention properties of SAMs, making it suitable for applications such as sand fixation.

Chemical modifications significantly enhance the performance of SAMs prepared from Pal. For example, acid modification removes soluble impurities from natural Pal and exposes more silanol groups on its surface, thus enhancing reaction activity. Consequently, the water absorption rate of PAA/Pal SAM made with acid-activated Pal (using 2 mol/L HCl solution) can reach up to 1241 g/g, a 94.2% improvement over untreated clay [101]. Heat activation also boosts performance by generating additional broken bonds on the Pal surface, enhancing its reactivity. Combining thermally activated Pal with PAA increases water absorbency to 1515 g/g, compared to just 639 g/g for the untreated clay-based composite [101]. Similarly, acid and heat treatments improve the water absorbency of PAM/Pal composites by modifying their chemical composition, crystalline structure, cation exchange capacity, and specific surface area. Incorporating heat-activated Pal (HPal) (at 400 °C) or acid-activated Pal (APal) into the PAM network can boost maximum water absorbency to 1964 g/g and 1715 g/g, respectively, significantly higher than that of raw-Pal-based composites. Modification with various salts changes the surface charge and properties of Pal differently. Notably, AlCl_3_ and FeCl_3_ modifications increase specific surface area while reducing cation exchange capacity. Although this does not significantly enhance water absorbency, it improves the repeated swelling ability of the SAM. The SAMs prepared with CaCl_2_-, AlCl_3_-, and FeCl_3_-treated Pal retain 81%, 82%, and 79% of their initial water absorbency, respectively, after five cycles of swelling–deswelling–reswelling tests [110]. The SAM embedded with Pal exhibits a porous surface, while the incorporation of Al^3+^-modified palygorskite (Al^3+^-Pal) leads to the formation of a planar surface. This change is due to the auxiliary crosslinking effect of Al^3+^, which is beneficial for enhancing the network strength [111]. Increasing the Al^3+^-Pal content from 0 to 20 wt% raises equilibrium water absorbency in deionized water from 475 to 1158 g/g, with a subsequent slight decrease to 695 g/g at 40 wt%. The presence of Al^3+^ ions aids in assistant crosslinking, boosting water absorbency and gel strength.

Organic modification further improves surface hydrophilicity, hydrophobicity, and particle dispersibility, leading to more uniform polymer nanocomposites. Modifying Pal with varying amounts of hexadecyltrimethylammonium bromide (HDTMA), a SAM prepared with an organification degree of 8.02 wt%, shows optimal performance. Incorporating 10 wt% HDTMA-Pal into the composite enhances water absorbency from 996 to 1282 g/g in deionized water and from 63 to 68 g/g in 0.9 wt% NaCl solution, with a notable improvement in the initial swelling rate [109]. Comparative studies indicate that SAM prepared using organically modified and thermally activated Pal exhibit superior water absorption performance.

Halloysite (HSY) is a well-crystallized mineral featuring hollow micro and nanotube multilayer structures and with a low surface hydroxyl density [74]. The use of HSY in polymer composites is becoming an increasing research focus, and it has been utilized as a novel additive for preparing SAMs [113]. Utilizing the reactivity of HSY, a poly(acrylic acid-co-acrylamide)/HSY (PAA-AM/HSY) SAM was synthesized using solution polymerization, with HSY serving as the inorganic component [114]. The research indicates that adding 10% HSY can significantly increase the water absorbency to 1276 g/g within 60 min, due to the uniform distribution of HSY in the polymer matrix and formation of pores with an average size of 10 μm. After five reuse cycles, 78% of its initial water absorption capacity was retained, proving the positive role of HSY in improving water absorption capacity and reusability.

### 4.2. Natural Polymers/1D Nanoclay SAMs

With the increasing attention to resource and environmental issues, the environmental friendliness of materials has become an important indicator for evaluating their applicability in practice [115]. In order to make SAMs safer and more environmentally friendly and improve their performance, various natural polymers or biomass are introduced into their structure [115]. Cellulose and chitosan (CTS) (deacetylated derivatives of chitin) are preferred renewable organic components for developing hybrid materials due to their low cost, abundance, biocompatibility, and biodegradability. Composites of natural polysaccharides and inorganic clay minerals represent a family of natural SAMs [116]. A large number of SAMs based on natural polymers such as starch [117], cellulose [118,119], guar gum [120], gelatin [121], carrageenan [122], sodium alginate [123], cashew gum [124], dextrin [125], and xanthan gum [126] have been developed. The addition of polysaccharides and inorganic clay minerals in SAMs enhances their performance and significantly reduces production costs [127].

Many SAMs based on natural starch, cellulose, CTS, gelatin, dextrin, alginate, and wheat bran have been developed, and the resulting materials show satisfactory performance and environmentally friendly characteristics. Aqueous solution polymerization, a green polymerization method, has been widely used to prepare SAMs based on natural polymers and clay minerals. Pal can be compatible with a variety of natural polymers and grafted polymers to form nanocomposites. Natural Pal can be directly used to prepare SAMs (Table 2) [128,129,130,131,132,133,134,135,136,137,138,139,140,141,142,143,144]. Using N, N’-methylene-bis-acrylamide (MBA) as a crosslinking agent, a novel chitosan-g-poly(acrylic acid)/Pal (CTS-g-PAA/Pal) SAM was prepared by grafting polymerization of CTS, AA, and Pal in aqueous solution, and its water absorption rate in distilled water was 159.6 g/g. Water absorption in 0.9 wt% NaCl solution reached 42.3 g/g. The results of structural analysis show that –OH of Pal, –OH of CTS, –NHCO, and –NH_2_ are involved in grafting polymerization with AA. The introduction of Pal enhances the thermal stability of the CTS-g-PAA SAMs and forms a loose and porous surface. The introduction of a small amount of Pal also improved the water absorption of CTS-g-PAA [128]. Kenawy et al. prepared CTS-g-PAM/Pal SAM by grafting polymerization and crosslinking of CTS, AM, and Pal. The results showed that when the amount of Pal increased to 0.4 g, the water absorbency increased from 210 to 319 g/g, and the thermal stability of the composite was also improved. However, as the clay content further increased to 1.2 g, the swelling capacity decreased to 170 g/g. When the pH value rose to 8, the water absorbency increased sharply. The slow-release results of urea fertilizer showed that when the Pal content of the prepared SAM increased from 0 to 1.2 g, the release rate was delayed, and the release time increased from 5 to 21 h [129].

**Figure 5:**
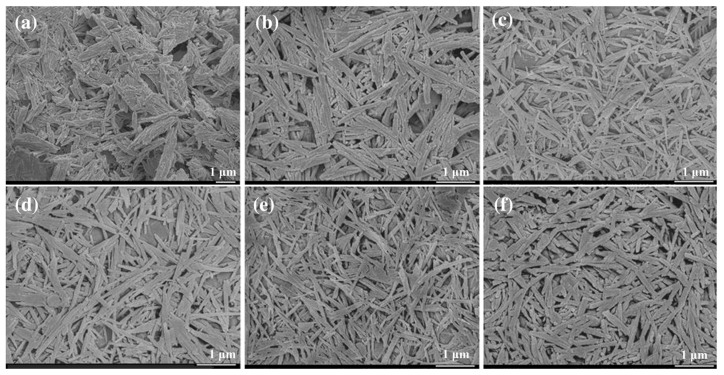
FESEM images of (**a**) natural palygorskite, (**b**) the unhomogenized Pal dispersed in water, and the homogenized Pal dispersed in an ethanol–water mixture with ratios of (**c**) 0:10, (**d**) 4:6, (**e**) 6:4, and (**f**) 10:0 [146]. Reprinted with permission from Elsevier.

Generally, Pal rod crystals in natural Pal clays usually exist in the form of crystal bundles or aggregates, so they cannot exert their special properties as 1D nanomaterials. For this reason, highly dispersed nanorods are expected to be obtained by the dissociation and dispersion of rod crystal bundles, thus improving their ability to enhance the performance of polymer materials. Conventional mechanical treatment methods, such as extrusion, high-speed shear, and grinding, cannot fully disaggregate the crystal beam at low force, and will destroy the rod-like crystal at high acting force, thus reducing the aspect ratio of the nanorods. In order to achieve lossless disaggregation of the rod crystal bundle, a high-pressure homogenization technique was employed by Xu et al. [145]. The hole effect of the high-pressure homogenization would generate inside-out stress inside the rod crystal bundle, and then split the crystal bundle into nanorods with high efficiency and low loss. In order to solve the problem of secondary agglomeration of disaggregated rod crystals, a new strategy of treating Pal with a mixed solvent of ethanol/water was employed (Figure 5) [146], which weakened the interaction between the rod-like crystals, thereby effectively improving the dispersion of the rod-like crystals without affecting their surface properties. This enabled the successful preparation of highly dispersed Pal nanorods.

High-pressure homogenization using various ethanol/water ratios led to effective dispersion of Pal, significantly affecting the strength and swelling properties of SAMs (SA-g-P(NaA-co-St)/Pal) prepared with it. Homogenization at 50 MPa with a 50% ethanol solution resulted in significant improvements: the gel strength increased from 1300 to 1410 Pa (at ω = 100 rad/s), the swelling capacity from 442 to 521 g/g, and the swelling rate from 3.3303 to 4.5736 g/g·s, and the re-swelling capacity of the SAM was enhanced. This confirms that the disaggregation of Pal crystal bundles is beneficial to improving the properties of the SAM [130].

Natural SPT can be directly used to synthesize natural polymer grafted polymer/clay SAMs. Santiago et al. [147] synthesized a series of SAMs through the polymerization and crosslinking of AA, SPT, and MBA in an aqueous solution. Results indicate that both the SPT content and its dispersion within the monomer significantly affect the water-absorbing performance of the SAM. When the SPT content is 5%, the water absorbency of the SAM in deionized water can reach 1419 g/g. The water absorption rate in salt solution (0.2 wt% NaCl solution) can reach 210 g/g. After the modification, the surface activity and dispersibility of the SPT will be significantly improved, and the properties of the SAM will be enhanced. Xie et al. [148] utilized CTS, AA, and SPT (including modifications like acid-activated SPT and cation-exchanged SPT) as primary materials to prepare CTS-g-PAA/SPT SAM via free radical grafting polymerization and crosslinking in an aqueous solution. The results showed that after adding 5% acid-activated SPT and Al^3+^-exchanged SPT, the water absorption rate of the prepared SAM increased by 18.7% and 40.6%, respectively, the water absorption rate constant increased by 113.2% and 317.2%, respectively, and the water absorption rate remained high, in the pH 4–12 range.

The comparative analysis of the results presented in Table 1 and Table 2 highlights significant differences in performance and environmental appeal between synthetic polymers and those of natural origin. Table 1 illustrates that synthetic polymer/Pal SAMs exhibit impressive water absorption capacities, reaching up to 396~2800 g/g in deionized water for PAM/organo-Pal composites [94]. However, these materials often face challenges related to biodegradability and environmental impact. Conversely, Table 2 demonstrates that natural-polymer-based SAMs can achieve water absorption capacities of 159.6~1317 g/g in distilled water (lower than that of synthetic-polymer-based SAMs), offering substantial advantages in terms of biodegradability and reduced environmental burden. This distinction underscores the critical importance of considering environmental appeal in the development of SAMs, particularly when aiming for sustainable agricultural practices. The use of natural polymers not only addresses concerns regarding long-term environmental impact but also promotes resource efficiency by utilizing renewable materials.

## 5. Role of 1D Nanoclays in SAMs

The advantages of 1D nanoclays, including a wide range of sources, low cost, high surface activity, good thermal stability, and strong mechanical properties, make them potential candidates in the development of SAMs with improved performance at reduced cost. Nanoclays are compatible with various polymer matrices, and can exist in SAMs in three possible ways [149,150,151]: (a) acting as crosslinking points and chemically bonding with polymers, with a nonlinear dependence relationship between water absorption rate and mineral particle size; (b) serving as endpoints for chemical bonding with polymers; and (c) being physically filled in the polymer network. The improvement of water absorbency by the introduction of nanoclay is mainly achieved by regulating the network structure.

SAMs outperform traditional absorbents because their hydrophilic network structure can retain significant amounts of water. When clay nanoparticles are introduced into the polymer matrix, they can regulate the regularity of the polymer network, preventing excessive polymer chain entanglement or polymer network collapse, and therefore increasing the water absorption capacity. At the same time, the polymer network is more likely to stretch when exposed to water after the clay particles are introduced, so the water absorption rate is usually increased. SAM was also prepared by graft copolymerization of starch phosphate, AM, and Pal; the introduction of 1D nanoclay changed the network composition and surface roughness, and the water absorption of 10 wt% Pal reached an optimum value of 1268 g/g when the mass ratio of AM to starch phosphate was 5:1, while the thermal stability and salt resistance of the SAMs were also enhanced [133]. Clay incorporation improves the water absorption capacity and rate of SAMs, which is beneficial for their application in agroforestry. Numerous studies have demonstrated that the introduction of clay minerals into the polymer matrix improves the water absorption multiplicity and rate in most cases, which undoubtedly provides a new and effective way to develop SAMs with better properties.

Nanoclay can be exfoliated into nanolayers in the polymer matrix, and its surface silanol groups can have strong interactions with hydrophilic groups on the polymer chains, so nanoclay chemically bonded or tightly physically crosslinked to the polymer chains usually improves the mechanical properties. Strong interfacial interactions between the polymer matrix and the nanoclay layer improve the mechanical strength, barrier, and thermal stability properties of the complexes [152]. When the content of clay minerals in SAMs is high, the clay particles exist as physical fillers in the SAP network, and then the clay’s contribution to the mechanical strength of the polymer network is limited, and even reduces the gel strength of SAMs. The influence of nanoclay on the mechanical properties of polymer networks mainly depends on the structure and morphology of nanoclay, its content, its size, and its existence state in the polymer matrix.

## 6. Synthesis Methods of SAMs Based on Clay

The various SAMs usually correspond to different preparation methods due to differences in raw materials, dispersion media, reaction conditions, and initiation methods. Today, SAMs are usually made using one of the following main methods: propriety polymerization, suspension polymerization, solution polymerization, and irradiation polymerization. Since the first SAM was reported by the U.S. Department of Agriculture [153], the research and development of SAMs have attracted great attention worldwide, and the products are widely used in many fields. Several polymerization methods such as aqueous solution polymerization [154,155], reverse suspension polymerization [156], microwave-initiated polymerization [157], the semi-dry method [97], and glow-discharge electrolytic plasma polymerization [158] have been used for the preparation of SAMs, with aqueous solution polymerization being the most widely employed. By using a similar procedure, a number of polymer/clay SAMs were prepared and evaluated, and a comprehensive comparison of their water absorption was conducted.

### 6.1. Solution Polymerization

**Figure 6:**
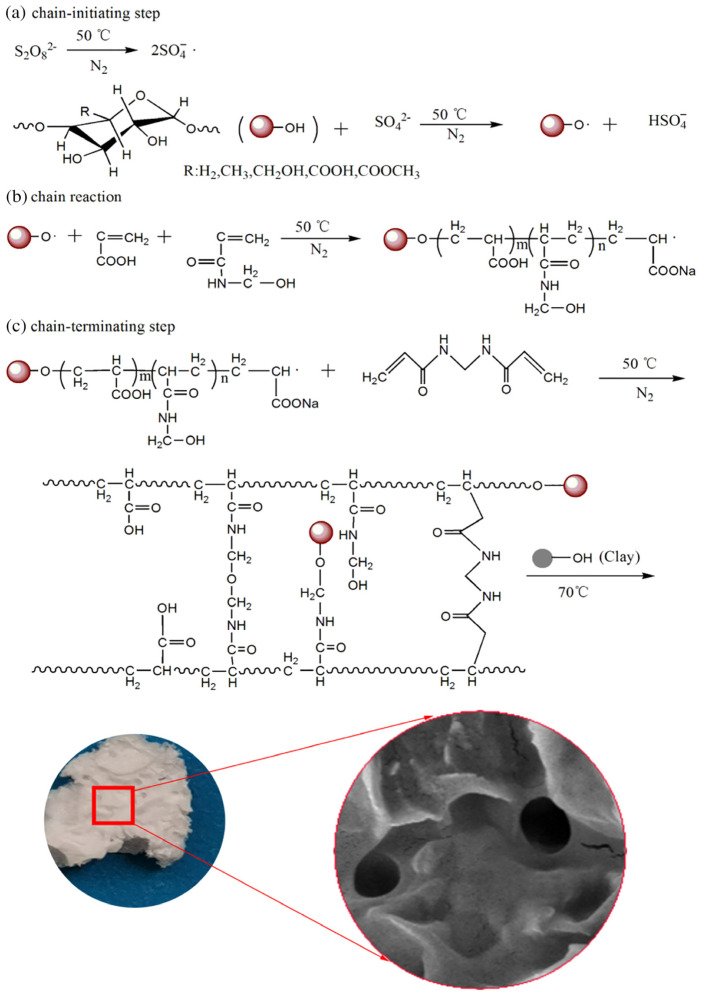
Scheme for the synthesis of natural polysaccharide-g-polymer/clay SAM through solution polymerization [91]. Reprinted with permission from the publisher.

Aqueous solution polymerization is a simple and green polymerization reaction process. In polymerization of vinyl monomer for the preparation of synthetic polymer/clay SAMs, the monomer, polymerization initiator, crosslinker, and inorganic particles (e.g., clay) can be dispersed homogeneously in a non-monomer liquid solvent, and then polymerized by redox initiation or thermal initiation methods to obtain a gel-like product. The reaction solvent is water, without the need to use a large number of organic solvents, which can greatly ensure safety and reduce production costs. The production process has low requirements for equipment, and the investment is thus obviously reduced. The reaction conditions are easy to control, and the production process does not produce pollution, which is conducive to the realization of pollution-free production. However, the aqueous solution polymerization must be carried out under a certain monomer concentration, and the polymerization rate is slow, the polymer molecular weight is low, and the production capacity is low at a low monomer concentration. For the preparation of natural polymer grafted polymer/clay SAM, the whole process is relatively complex. The polymerization reaction process is shown in Figure 6 [91]. It is necessary to dissolve the natural polymer in water or aqueous solution first to form a homogeneous solution, and then add an initiator (e.g., APS). In the initial stage, the initiator APS decomposes under heat to produce a high concentration of sulfate anion radicals. Subsequently, these radicals strip hydrogen from the –OH group of the natural polysaccharide to form large radicals, while the viscosity of the polysaccharide solution decreases. These macro radicals can act as active sites during the reaction and can initiate chain growth of the vinyl group of the monomer. During chain propagation, the crosslinker MBA with bis-vinyl groups participates in the polymerization reaction, while the clay particles bind to the polymer network through their reactive silanol groups, which interconnect the polymer chains to form a crosslinked network [91]. Due to the high viscosity of the reaction system in the presence of polysaccharides, the free radical migration is affected, so the natural polymer is usually reacted with the initiator first to form macromolecular free radicals, which can improve the grafting efficiency to some extent.

### 6.2. Suspension Polymerization

**Figure 7:**
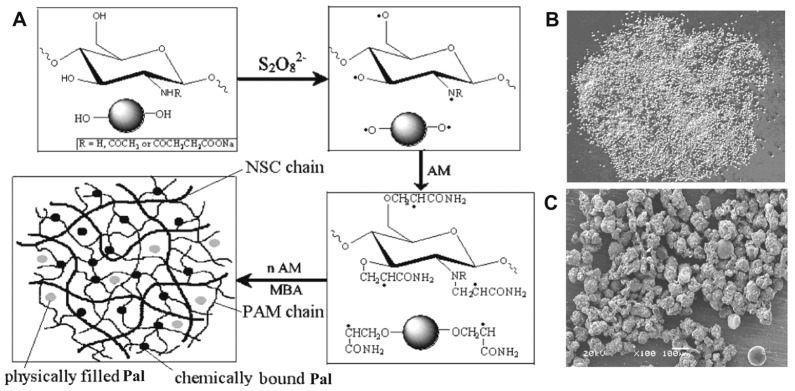
(**A**) Scheme illustrating the synthesis of the NSC-g-PAM/Pal SAM; (**B**) digital photo of the NSC-g-PAM/Pal SAM; (**C**) SEM image of NSC-g-PAM/Pal [159]. Reprinted with permission from the publisher.

Suspension polymerization, also called pearl, bead, or particle polymerization, utilizes the differences in hydrophilicity and hydrophobicity among monomers. This method involves mechanically agitating monomers or their mixtures into a liquid phase like water, allowing polymerization of the monomer droplets while continuously dispersing them. In this process, an aqueous solution is typically used as the dispersed phase, and a water-insoluble hydrocarbon solvent is used as the continuous phase. Water-soluble monomers or other polymer matrices are dissolved or suspended in the aqueous phase as droplets. Following initiation, the polymerization occurs within these aqueous droplets, forming polymer particles or microspheres with diameters ranging from 0.01 to 10 mm directly during the reaction. For example, by suspension polymerization using water as the dispersed phase and heptane as the continuous phase, granular N-succinimidyl chitosan-g-polyacrylamide/palygorskite (NSC-g-PAM/Pal) SAMs with improved properties can be formed [159]. During the reaction, chitosan in the dispersed phase first reacts with the initiator to form macromolecular radicals. These radicals, along with the initiator, then trigger the grafting and copolymerization of vinyl monomers onto the macromolecular chains. In the presence of crosslinking agents, these grafted and copolymerized chains interconnect to form a 3D network. In this process, part of the clay participates in the crosslinking reaction through the silanol groups on the surface, and most of the clay particles exist in the polymer network in the form of physical fillers, which interact with the polymer chains by hydrogen bonding or van der Waals forces, to obtain stable nanocomposite networks (Figure 7) [159]. Liu et al. used reverse-phase suspension polymerization to copolymerize acrylic acid, acrylamide with HSY, and graphene for copolymerization and crosslinking to obtain poly(acrylic acid-co-acrylamide)/halloysite/graphene SAMs [160]. Compared with the traditional aqueous solution polymerization method, the SAMs obtained by reversed-phase suspension polymerization are usually spherical, and their optimal water absorbency can reach 743.9 g/g. The rate of water absorption is significantly when the contents of both HSY and GO are 0.1%, with the rate constant increasing from 2.11 to 9.14 g/min. Reversed-phase suspension polymerization offers several advantages: it lowers the viscosity of the reaction system, stabilizes the polymerization reaction, and increases the reaction rate constant from 2.11 to 9.14 g/min. The reaction system has low viscosity, the polymerization reaction is stable, the reaction heat can be easily dissipated, the product particles are uniform, and the post-processing of the product is very simple. However, this method also has some disadvantages: there are some dispersants in the product, so the organic solvents need to be recycled, which requires a large investment; it may cause environmental pollution to a certain extent. The suspension process is implemented by only a few companies, as it offers a higher degree of production and product engineering control during the polymerization step.

Due to the good dispersibility of HSY in liquids, a poly(acrylic acid-co-acrylamide)/carboxyl-modified HSY–graphene oxide (PAA-co-AM/HSY–GO) SAM was synthesized by inverse suspension polymerization. It was found that HSY and GO can be uniformly dispersed in the PAA-co-AM copolymer matrix and have a synergistic effect, significantly improving water absorption and retention capacity [160]. The uniform dispersion of HSY in polymer matrix and its inhibitory effect on disordered entanglement of polymer chains contribute to significantly improving the water absorption performance of SAMs. However, due to the low natural reserves of HSY and the high cost of synthetic HSY, the research and industrial development of SAMs based on HSY are relatively slow.

### 6.3. Emulsion Polymerization

**Figure 8:**
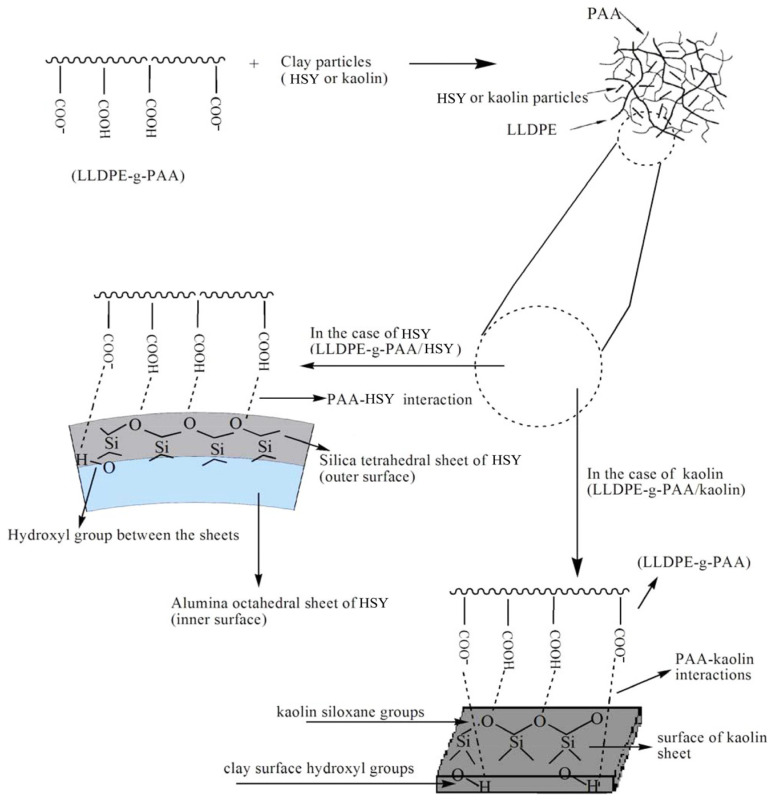
Structure of LLDPE-g-PAA/HSY or Kaolin [161]. Reprinted with permission from the publisher.

Emulsion polymerization involves forming an emulsion of monomers in water and using an emulsifier under mechanical agitation or vibration to produce latex, powder, or needle-shaped polymers. Two types of SAMs were prepared by emulsion polymerization using waste linear low-density polyethylene (LLDPE), AA, and two types of clay (including kaolinite and HSY) as raw materials. The preparation process involves dissolving 1 g of waste LLDPE in 20 milliliters of toluene in a four-necked reaction flask equipped with a thermometer and mechanical stirrer. This is done at 90 °C under a nitrogen flow to remove oxygen from the system. Next, the temperature is lowered to 60 °C, and 0.3 g of Span 60 (as an emulsifier) is added to the solution. Then, a mixture containing partially neutralized AA and either HSY or kaolinite powder (0.3, 0.5, 0.7, 0.9, or 1.1 g) is added. After 10 min, an appropriate amount of crosslinking agent MBA is added to the solution. Upon reaching the gel point, the temperature is increased to 70 °C and maintained for 3 h. The sample is then washed with ethanol, cut into small pieces, and dried at 80 °C for 12 h to obtain the final product, whose structure is shown in Figure 8 [161]. Compared with the hydrogel without clay, the SAM has more pores and a higher swelling ratio, and the introduction of 5 wt.% of clay can significantly improve its water absorption capacity by 26.66% [161].

### 6.4. Decentralized Polymerization

CTS possesses highly reactive hydroxyl and amino groups, which can be readily modified through various chemical and physical methods for the preparation of SAMs. Unlike other polysaccharides, granular CTS-based SAMs can be directly formed in an aqueous solution by controlling the reaction conditions. Among natural polymers, CTS is unique as a cationic polysaccharide containing an amine group, enabling stronger hydrogen-bonding interactions with PAA chains produced from AA polymerization. In the presence of crosslinking agents, CTS-g-PAA polymers may become entangled to form granular products, and the introduction of palygorskite makes it easy to form granular products [128]. This is the first report on the preparation of granular SAMs by aqueous solution polymerization reaction, which opens up a new way to prepare novel granular SAMs in a one-step reaction. The formation mechanism of the granular product primarily involves the electrostatic assembly of positively charged CTS with negatively charged acrylic acid, along with associated grafting and crosslinking reactions. The –NH_2_ group of CTS can be protonated by the H^+^ of acrylic acid, which produces electrostatic interactions with the –COO^−^ group to form the –NH_3_^+^⋯^−^OOC– pair. Following polymerization initiation, acrylic acid monomers are grafted onto the CTS backbone to form PAA chains. These chains intertwine with the CTS macromolecular chains, leading to the formation of numerous insoluble particles. In the presence of a crosslinking agent, the grafted chains can crosslink to form a network structure, and these particles can aggregate with each other to form a granular product.

### 6.5. Polymerization by Irradiation

High-energy radiation, such as through gamma rays and electron beams, is emerging as a green method for preparing clay-based SAMs. Unlike conventional methods, high-energy radiation can generate free radicals like hydroxyl radicals, initiating polymerization and crosslinking reactions to form polymer networks with minimal use of chemical initiators. Compared with the traditional polymerization method, the pore structure of the SAMs prepared by ^60^Coγ irradiation initiation was better than that of the chemical initiator. ^60^Coγ rays initiate the reaction of acrylic acid grafting onto the SA skeleton belonging to the free radical reaction. Most of the irradiation energy is absorbed by water, generating hydroxyl radicals. These radicals excite the double bonds of AA, causing them to break and form new radicals, initiating the homopolymerization of AA. At the same time, the hydroxyl radical attacks the SA, causing the C–H bond to break to form an SA-based radical, which then reacts with the acrylic acid molecule and then propagates, leading to chain growth and subsequent network formation by crosslinking. In addition to gamma rays, glow discharge has also been used to synthesize SAMs.

Compared with the traditional chemical initiation method, the ray initiation is cleaner and does not introduce chemicals into the material; moreover, the initiation efficiency is higher, which is conducive to obtaining products with better performance. The disadvantage is that ray initiation requires a large amount of equipment and is difficult to apply on a large scale.

### 6.6. Microwave-Initiated Polymerization

Partially neutralized AMPS, MBA, and Pal were polymerized via microwave-radiation-induced free radical grafting to create salt-resistant SAMs. The impact of microwave power and Pal content on water absorption was examined. Under microwave initiation, chemical/physical interactions between Pal and polymer chains were generated. The introduction of an appropriate amount of Pal could effectively improve the water absorption and saline-resistant properties of the SAMs, and the water absorbency for deionized water and physiological saline was 986 and 102 g/g, respectively, at 5% clay content [162]. One study shows that a SAM can be synthesized via the aqueous solution polymerization of acrylic acid, acrylamide, and palygorskite clay under microwave irradiation [163]. The preparation process of SAM takes only 30 s, demonstrating fast reaction kinetics. It was observed that microwave irradiation time, the neutralization degree of acrylic acid, initiator concentration, crosslinker dosage, and the amount of Pal significantly influence the performance of the resultant SAMs. The SAM exhibits an absorption capacity of approximately 1620 g/g in distilled water and around 170 g/g in a 0.9 wt% NaCl solution.

## 7. Applications of SAMs Based on 1D Nanoclay

SAMs based on 1D nanoclays have been extensively utilized in many fields such as agriculture, forestry, drug delivery, and wastewater treatment for their superior water absorption and retention capabilities, as well as their lower cost. The special 3D network structure of SAMs allow them to store water or drugs, achieving drug delivery, and the abundant functional groups of SAMs make them capable of capturing various ions or molecules to achieve decontamination of wastewater. This section introduces the representative applications of 1D-nanoclay-based SAMs.

### 7.1. Agriculture and Forestry

SAMs can help to improve soil structure and enhance water-holding capacity, thereby promoting plant growth and significant improvements in crop yields under both normal and drought conditions [164,165,166]. A study showed that the hydroxypropyl methylcellulose-g-PAA/polyaspartic acid/Pal (HPMC-g-P(AA-co-PASP)/ATP) SAM can prevent water leakage in soils [167]. It achieved maximum equilibrium absorption rates of 1785 g/g in deionized water, 254 g/g in tap water, and 138 g/g in a 0.9 wt% NaCl solution. Its application to soil significantly improved water holding and retention capacities, and reduced leaching of added urea and decreased water permeability in treated soils. Another study also confirmed that the wheat straw-g-PAA/Pal (water absorption capacity: 186 g/g in tap water) SAM can be used to produce a slow-release nitrogen (N) and boron (B) fertilizer with water-retention properties, which can enhance fertilizer use efficiency and reduce environmental impact [144]. The product, containing 23.3% nitrogen and 0.65% boron, not only retains water effectively but also releases nutrients slowly. These studies suggest that SAMs containing 1D nanoclay hold potential for advancing sustainable agricultural practices.

### 7.2. Drug Delivery Vehicles

Drug delivery systems have garnered significant attention due to their capability to efficiently deliver medication to targeted areas within the body. Clay-based SAMs have found applications as drug carriers due to their unique structural features and biocompatibility. This drug delivery system allows for a sustained release of active compounds, enhancing therapeutic efficacy. Such systems not only enhance drug efficacy but also minimize side effects and reduce the frequency of dosing by extending the duration of drug release. The SAP beads made from CTS-g-PAA/Pal and sodium alginate (SA) were proved to be effective as drug delivery matrices. These matrices are crosslinked via Ca^2+^ ions, leveraging the ionic gelation properties of SA. The resulting SAP beads exhibit notable pH sensitivity. When tested for the release of diclofenac sodium (DS), these beads released only 3.76% of DS in a pH 2.1 solution over 24 h, while achieving complete release in a pH 6.8 solution within the same timeframe. Notably, at a pH of 7.4, full release was accomplished within just 2 h. Incorporating 10% Pal into the formulation was found to prolong the drug release duration [168]. Furthermore, a SAP bead composed of GG-g-PAA/Pal and SA was synthesized, showcasing excellent pH sensitivity. In vitro release kinetics using DS as a model drug demonstrated that the addition of Pal significantly mitigated the burst release effect of DS, leading to a clear reduction in the cumulative release of DS with increased Pal content [169].

### 7.3. Wastewater Treatment

Various pollutants such as dyes, heavy metals, antibiotics, oils, and others are commonly found in natural water bodies, leading to increasingly severe water pollution that threatens ecosystem safety and human health [170,171,172,173,174,175,176]. Adsorption has long been an effective method for purifying water of contaminants due to its simplicity, low energy consumption, high efficiency, and thorough removal capabilities towards pollutants, making it widely used in water treatment [177]. Among available adsorbents, materials with a 3D network structure exhibit high adsorption capacity and a rapid adsorption rate. Studies have shown that SAMs can adsorb dye pollutants with extremely high capacity due to their electrostatic attraction and chemical interaction with organic molecules. The P(AA-co-AM)/Pal SAM achieves a maximum adsorption capacity of 1194 mg/g for Methyl Violet [178]. A P(AA-co-IA)/Pal composite effectively adsorbs malachite green, with the addition of 5 wt% Pal enhancing both the adsorption rate and capacity to 2433 mg/g [179]. The CTS-g-PAA/Pal composites showed satisfactory adsorption capacity and increased the adsorption rate for Cu^2+^ [180], and the composites with 10, 20, and 30 wt% Pal were fast-acting, with more than 90% of the maximum adsorption capacity for Cu^2+^ occurring within the initial 15 min. When Pal content exceeds 30 wt%, excess Pal acts as a filler in the polymeric network of the composite. With the inclusion of Pal, the adsorption capacity still reaches 170.65 mg/g, which is conducive to decreasing the cost of adsorbent. These studies also revealed that the SAM can adsorb heavy metals by the synergy of electrostatic interaction, chemical complexing of functional groups (e.g., –NH_2_, –OH, and –COOH groups), network holding, and ionic exchange. Of these, chemical complexing is the primary driving force for adsorption. As for the NH_4_^+^ pollutant that is difficult to purify, the CTS-g-PAA/Pal SAM can adsorb ammonium nitrogen at 21.0 mg NH_4_^+^–N per gram when containing 20 wt.% Pal [181]. Adsorbed NH_4_^+^–N can be fully desorbed by 0.1 mol/L NaOH within 10 min. Adsorption of NH_4_^+^–N onto CTS-g-PAA/Pal occurs very rapidly, achieving over 90% adsorption within 5 min. Incorporating 50 wt.% Pal decreases the adsorption capacity slightly from the maximum of 21.0 mg/g (at 20 wt.% Pal) but significantly reduces costs. Adding 20% Pal enhances the adsorption capacity from 19.9 to 21.0 mg/g. The 3D network structure of the SAM contributes to adsorption capacity, but the presence of –COO^−^ groups within the polymeric network plays a more critical role. Electrostatic attraction between –COO^−^ sites and NH_4_^+^ ions primarily controls the adsorption process. An appropriate amount of Pal increases surface area and porosity, enhancing adsorption capacity. However, excessive Pal can reduce hydrophilic –COO^−^ group proportions, leading to decreased adsorption efficiency. In summary, SAP composites offer a promising solution for NH_4_^+^ removal, due to their structural characteristics and the presence of functional groups that facilitate efficient adsorption.

## 8. Trends and Prospects for Clay-Based SAMs

Currently, most clay-based SAMs are used in agriculture, forestry, and ecological restoration due to their superior water absorption properties and lower costs. However, conventional superabsorbent polymers (SAPs), such as polyacrylates, suffer from high production costs and limited salt resistance, which significantly reduces their effectiveness in practical applications like agriculture where soil salinity is a common issue. Clay-based SAMs address these limitations by offering enhanced salt tolerance and lower costs. Increasing the rate of water absorption, reducing the cost, and utilizing novel synthetic methods to prepare SAMs continue to constitute an interesting area of research. In recent years, the cross-fertilization of chemistry, mineralogy, and material science has led to significant development of synthesis technology; in particular, the development of modification technology of minerals has made it possible to enhance their performance, which implies that SAMs can be prepared with modified minerals to further enhance their overall performance. Designing and developing new SAMs and their industrialization have become crucial for determining market prospects. According to the current research on SAMs, their further development includes the following aspects.

(1) Developing new types of SAMs: For applications in agriculture, liquid hydrophilic polymers can be designed and developed, which can bind to soil particles after entering the soil to improve water-holding performance, which is not limited by differential osmotic pressure. The water retention properties of liquid water-holding materials are not affected by external ions or salinity, making them more suitable for saline and sandy soils. In addition, using waste to develop SAMs also shows great prospects, because it involves harmless disposal and value-added utilization of waste. Compared with traditional polymer-based SAMs, these approaches reduce production costs and minimize environmental impact.

(2) Developing multifunctional SAMs: In order to meet the demands of different scenarios, it is necessary to ensure that SAMs have excellent water absorption capacity, water absorption rate, good gel strength, and salt resistance. However, almost all of the SAMs developed so far cannot meet these requirements. In general, SAMs for agriculture need to have excellent salt resistance, pH stability, reusability, and fertilizer release properties. Salt resistance, gel strength, water absorption rate, and multifunctionality of SAMs have become bottlenecks, restricting the development and application of SAMs, as well as creating challenges for material designers. Therefore, SAMs with special structures and compositions should be designed, and new preparation processes should be developed in the future.

(3) Improving the environmental friendliness of SAMs: As SAMs continue to be popularized, the need for such materials to be environmentally friendly has become an important factor affecting large-scale use in the future. The design and synthesis of biodegradable SAMs with excellent properties is a crucial future direction. Among the many possible approaches, the manufacture of biodegradable SAMs from raw materials available in nature remains the most promising method. Cellulose is the world’s most abundant biodegradable polymer, starch is a readily available polysaccharide compound that is widely found in plants such as corn, potatoes, and sweet potatoes, and amino acids contain many hydrophilic carboxyl and amino groups that can be combined with clays to design eco-friendly SAMs. Compared to conventional synthetic polymers, natural materials provide a sustainable and eco-friendly option for SAM production, addressing both environmental concerns and resource utilization efficiency.

## 9. Summary

The unique properties and broad application prospects of SAMs have established them as one of the fastest-growing categories of polymer materials. Over the past few decades, SAMs have found applications ranging from agriculture and hygiene products to pharmaceuticals, wastewater treatment, and others. This expansion has driven demand and promises sustained growth in the SAM sector. Manufacturers are increasingly focused on enhancing performance while reducing costs through the development of various raw materials and methods aimed at translating research findings into industrial production. In this context, we summarize the main types of 1D clays such as Pal, HSY, and SPT, and their incorporation to enhance the performance of SAMs, such as in water absorption capacity and rate and water retention. Addressing the issue of high cost associated with traditional SAMs, these clays improve overall performance and reduce cost. Additionally, we highlight the primary types of polymers used for subsequent modification, which include both synthetic and natural polymers. The combination of natural polymers with clays significantly improves environmental friendliness. The choice of polymerization method is crucial for achieving optimal performance and cost-effectiveness. Among various techniques—such as proprietary, suspension, and solution polymerization—solution polymerization stands out for its simplicity and suitability for industrial-scale production. The advantages of these polymerization methods lie in their ability to improve efficiency and reduce costs, driving advancements in SAM technology. Future progress in the SAM industry will depend on continued improvements in polymerization technologies and manufacturing processes, aiming to lower costs and boost performance further. This summary encapsulates the key aspects of our research, providing a comprehensive overview of the types of 1D clays, polymers, and polymerization methods pivotal to the development and industrial application of SAMs.

## Figures and Tables

**Table 1:** Comparison of the water absorbency (WA) of different SAMs based on Pal and modified Pal (MPal).

SAMs	Clay Content (%)	Swelling Medium	WA (g/g)	Ref.
MPal/PASP	2	Deionized water	892	[96]
MPal/PASP	2	0.9 wt.% NaCl solution	95	[96]
PAA/Pal	35	0.9 wt.% NaCl solution	98.2	[97]
PAA/Pal	5	Deionized water	1325	[95]
PAA/Pal	5	0.9 wt.% NaCl solution	117	[95]
PAA/Pal	10	Deionized water	1017	[95]
PAA/Pal	10	0.9 wt.% NaCl solution	77	[95]
PAM/HPal	10	Deionized water	1964	[98]
PAM/APal	10	Deionized water	1469	[98]
P(AA-co-AMPS)/Pal	8.76	0.9 wt.% NaCl solution	47	[99]
P(AA-co-AMP)/Pal	8.48	Deionized water	427	[100]
P(AA-co-AMP)/Pal	30	Deionized water	396	[100]
PAM/APal	10	Deionized water	2140	[94]
PAM/APal	10	0.9 wt.% NaCl solution	100	[94]
PAM/HDTMABr-Pal	10	Deionized water	2800	[94]
PAM/HDTMA-Pal	10	0.9 wt.% NaCl solution	121	[94]
PAA/HPal	30	Deionized water	1515	[101]
PAM/HDTMA-Pal	10	Deionized water	1874	[102]
PAM/HDTMA-Pal	20	Deionized water	1319	[102]
PAM/Pal	30	Deionized water	2037	[103]
PAA/STMACl-Pal	10	Deionized water	562.1	[104]
PAM/Pal	30	Deionized water	1715	[105]
PAM/Pal	30	0.9 wt.% NaCl solution	87.8	[105]
PAA-AM/Pal	10	Deionized water	1400	[106]
PAA-AM/Pal	10	0.9 wt.% NaCl solution	110	[106]
PAA/Pal/SH	20	Deionized water	583	[107]
PAA/Pal/SH	20	0.9 wt.% NaCl solution	63	[107]
PAA-AM/SH/Pal	10	Deionized water	996	[108]
PAA-AM/SH/Pal	10	0.9 wt.% NaCl solution	63	[108]
PAA-AM/O-Pal/SH	10	Deionized water	1282	[109]
PAA-AM/O-Pal/SH	10	0.9 wt.% NaCl solution	68	[109]
PAA/Cl^−^-Pal	30.4	Deionized water	675	[110]
PAA/Cl^−^-Pal	30.4	0.9 wt.% NaCl solution	70	[110]
PAA/Al^3+^-Pal	20	Deionized water	1158	[111]
PAA/Al^3+^-Pal	40	Deionized water	695	[111]

**Table 2:** Summary of the water absorbency (WA) of different natural polymer/Pal SAMs.

SAMs	Clay Content (%)	Swelling Medium	WA (g/g)	Ref.
SA-g-P(NaA-co-St)/Pal	10	Distilled water	521	[130]
WB-g-PAA/Pal	3.94	Distilled water	513	[116]
GG-g-P(NaA-co-St)/Pal	10	Distilled water	530	[131]
GG-g-P(NaA-co-St)/Pal	10	0.9 wt.% NaCl solution	65	[131]
CMC-g-PAA/Pal	10	Distilled water	666.67	[132]
SA-g-poly(NaA-co-NaSS)/Pal	10	Distilled water	532	[133]
ST-g-PAM/Pal	10	Distilled water	1317	[134]
ST-g-PAM/Pal	10	0.9 wt.% NaCl solution	68	[134]
P-St-AM/Pal	10	Distilled water	1268	[135]
SA-g-PNaA/Pal	20	Distilled water	685	[136]
SA-g-poly(NaA-co-St)/Pal	10	Distilled water	587	[137]
SA-g-poly(NaA-co-St)/Pal	10	0.9 wt.% NaCl solution	73	[137]
CTS-g-PAA/Pal	2.5	Distilled water	159.6	[128]
CTS-g-PAA/Pal	2.5	0.9 wt.% NaCl solution	42.3	[128]
CTS-g-PAM/Pal	5.56	Distilled water	319	[129]
SCB-g-PAM/Pal	5.3	Distilled water	920	[138]
PSY-g-PAA/Pal	10	Distilled water	568	[139]
PSY-g-PAA/Pal	10	0.9 wt.% NaCl solution	64	[139]
SS-g-PAA/Pal	12	Distilled water	1236	[140]
SS-g-PAA/Pal	12	0.9 wt.% NaCl solution	108	[140]
CMC-g–PNaA/Pal	10	Distilled water	614	[141]
CMC-g-P(AA-AMPS)/Pal	18	Distilled water	864	[142]
CMC-g-P(AA-AMPS)/Pal	18	0.9 wt.% NaCl solution	72	[142]
HEC-g-PAA/Pal	5	Distilled water	421	[143]
CMWS-g-PAA/Pal	14	Distilled water	186	[144]

## Data Availability

Data are contained within the article.

## Abbreviations

The following abbreviations are used in this paper:

SAM	Superabsorbent material
SAMs	Superabsorbent materials
AA	Acrylic acid
AM	Acrylamide
PAA	Poly(acrylic acid)
PAM	Poly(acrylamide)
Pal	Palygorskite or attapulgite
HSY	Halloysite
SPT	Sepiolite
NSC-g-PAM/Pal	N-succinimidyl chitosan-g-polyacrylamide/palygorskite (or attapulgite)
HPal	Heat-activated palygorskite or attapulgite
APal	Acid-activated palygorskite or attapulgite
MPal	Modified palygorskite or attapulgite
AN	Acrylonitrile
AMPS	2-acrylamido-2-methylpropanesulfonic acid
GT	Tragacanth gum
SA	Sodium alginate
CMC	Carboxymethyl cellulose
LLDPE	Low-density polyethylene
GO	Graphene oxide
ST	Starch
WB	Wheat bran
CTS	Chitosan
St	Styrene
NaSS	Sodium styrenesulfonate
NaA	Sodium acrylate
MBA	N,N’-methylenebisacrylamide
APS	Ammonium persulfate
SCB	Sugarcane bagasse
WA	Water absorbency
